# Measuring Parental Behavior towards Children’s Use of Media and Screen-Devices: The Development and Psychometrical Properties of a Media Parenting Scale for Parents of School-Aged Children

**DOI:** 10.3390/ijerph18179178

**Published:** 2021-08-31

**Authors:** Kateřina Lukavská, Jaroslav Vacek, Ondřej Hrabec, Michal Božík, Michaela Slussareff, Martina Píšová, David Kocourek, Lucie Svobodová, Roman Gabrhelík

**Affiliations:** 1 Department of Addictology, General University Hospital in Prague, 12000 Prague, Czech Republic; jaroslav.vacek@lf1.cuni.cz (J.V.); roman.gabrhelik@lf1.cuni.cz (R.G.); 2 Department of Addictology, First Faculty of Medicine, Charles University, 12000 Prague, Czech Republic; 3 Department of Psychology, Faculty of Education, Charles University, 11000 Prague, Czech Republic; ondrej.hrabec@pedf.cuni.cz (O.H.); michal.bozik@vudpap.sk (M.B.); martinapisova3@gmail.com (M.P.); d.kocourek92@gmail.com (D.K.); zipkaa1@seznam.cz (L.S.); 4 Research Institute of Child Psychology and Pathopsychology, 831 05 Bratislava, Slovakia; 5 Institute of Information Studies and Librarianship, Faculty of Arts, Charles University, 11000 Prague, Czech Republic; Michaela.Slussareff@ff.cuni.cz

**Keywords:** media parenting, parental mediation, active mediation, restrictive mediation, media use, screens, psychometrics, self-report, inventory, measurement

## Abstract

Children’s excessive screen use is associated with health risks such as obesity, sleep problems, attention problems, and others. The effect of parental regulative efforts focused on screen/media use (media parenting) is currently unclear and difficult to examine given the heterogeneity of measuring tools used for its assessment. We aimed to develop an inventory that would enable reliable and valid measurement of media parenting practices (especially active and restrictive mediation) in parents of primary school children. The inventory builds on existing tools, it is comprehensive, yet easy to use in research setting. The original MEPA-36 (36 items) and revised MEPA-20 (20 items) inventories were examined using data from 341 Czech and Slovak parents of children aged between 6 and 10 years. Psychometrical properties were estimated using confirmatory factor and reliability analyses. Model fit was better for MEPA-20 and similar to other currently available tools. Both active and restrictive mediation subscales demonstrated high internal consistency. The internal consistency of newly constructed risky mediation subscales (risky active, risky restrictive, and over-protective mediation) was low. MEPA-20, especially active and restrictive mediation subscales, can be recommended for research on media parenting in context of screen/media use of school-aged children.

## 1. Introduction

Children’s use of screens has been an important topic of pediatrics, psychology, and other disciplines concerning children’s health and well-being since the mass spread of TV. Scholars have analyzed watching TV especially in relation with obesity (TV watching as a form of sedentary behavior) and violence (the possible negative outcomes of aggressive content). Today, we witness a mass spread of other screen devices such as smartphones, tablets, computers, gaming consoles etc. Children can use screens almost anytime, anywhere, and anyhow. As a result, children are spending an increasing amount of time with screens [1]. There is much evidence that the inappropriate use of screens may have serious negative outcomes for children. According to large population-based studies, children’s overuse of screens was found to be associated with obesity [2], sleep problems [3], higher level of emotional distress, depressive symptoms [4], attention problems [5], and other unfavorable conditions [6]. However, new information and communication technologies bring important benefits and are eminently indispensable, as the COVID-19 pandemic clearly has shown. Finding the balance between healthy and fruitful use and the harmful (over) use of screens is of the essence. Parental regulation of children use of media is very important given the fact that the new modern online media and online communication are nearly impossible to control by governments or by standard regulatory mechanisms [7]. Parenting in general, as a sum of practices and parental behavior toward a child, has been confirmed to affect many forms of adolescent risk or harmful behaviors (e.g., substance use, risky sexual behaviors, delinquency [8]), and also problematic internet use [9,10]. However, current evidence indicates that parenting strategies focused on media regulation (media parenting) are not very effective in preventing risky or problematic behaviors related to the use of media/screen-devices in children and adolescents [11,12,13].

### Media Parenting

The concept of media parenting, often called ‘parental mediation’ [7], is grounded in media consumption research, which has developed in the context of increasing television consumption in children during the second half of the last century. Despite the fact that there is not one predominant theory on parental mediation, scholars usually distinguish between two related but distinct factors: ‘active’ and ‘restrictive’ mediation. Active mediation (AM) originally reflected to what extent parents discussed the content of media with their child [14], but has been broadened to reflect general levels of communication about media and also shared experiences of media use between parent and a child [15]. Restrictive mediation (RM) reflects mostly parental practices of developing and implying regulative rules over child’s media use [7,15,16]. These two concepts (AM and RM) have been adopted by scholars focusing on new media such as the internet or games [11,13].

Surprisingly, media parenting (MP) has not shown consistent effects either on the intensity of children’s media use (i.e., media time) [11] or on the problematic use of modern media [13]. The meta-analysis by Collier et al. (2016) assessed the effect of three separate predictors—restrictive mediation, active mediation, and co-viewing—on media time (predominantly watching TV) and several other adolescent outcomes such as aggression, substance use, and sexual behavior. It seems that there is almost no relationship between media parenting and media use, except for the small but significant positive effect of co-viewing on media time, that was present for both watching TV and for online gaming. No effects were found of restrictive or active mediation on the use of internet or games, but, notably, there are a limited number of studies on modern media (eight studies on internet or gaming and RM, and two studies on internet/gaming and AM) [11]. A systematic literature review by Nielsen et al. (2019) focused on the relationship between media parenting and the problematic use of the internet (PIU) and problematic online gaming (POG). In the case of the relationship between RM and PIU, six studies demonstrated a negative association (i.e., protective effect), three studies showed no effect, and three studies demonstrated a positive association (i.e., promoting) effect of RM on PIU. Even more ambiguous results were obtained for POG—three studies suggested the protective effect, three studies showed no effect, and three studies suggested the promoting effect of restrictive mediation on POG. The results were similar for AM—six studies showed the negative association with PIU (i.e., suggested the protective effect), but four studies showed no effect, and one study showed the positive association (i.e., suggested the promoting effect). Most studies (three from four) on AM and POG found no effect [13]. These results may sound improbable and can even be frustrating—does it really not matter what parents do about their children’s use of media?

We believe that the inconclusive results are at least partially caused by methodological issues. We thoroughly analyzed the measurement of media parenting (active and restrictive mediation) and identified various problems. First, studies usually used a child’s report of parenting practices, which may or may not be very accurate. Second, most studies relied on measures of their own construction with various degree of psychometrical quality. Moreover, currently available measuring instruments usually focused only on one type of screen (e.g., smartphone) or on one type of media (e.g., internet), which may not appropriately reflect the reality of media parenting, which is usually focused rather globally on the “use of screens/all media” [17]. Most importantly, most instruments do not distinguish potentially effective and potentially counter-effective practices; for example, setting clear and appropriate rules specifying a child’s screen time may be a good practice of RM, while forcing a child to immediately quit the use of a screen device when a parent feels like child was using it for “too long” seems to be a rather negative example of RM.

Given the lack of quality and properly constructed measures of media parenting, we aimed to develop a comprehensive self-report inventory for parents of school-aged children, that would (1) focus on the use of screen devices/media in general, and (2) clearly distinguish between positive (effective) and negative (counter-effective or potentially harmful) practices of both active and restrictive mediation. This study describes the construction of the scale and presents the results of its pilot testing.

## 2. Materials and Methods

### 2.1. Setting and Data Collection

The target population were parents of children attending the first, second, or third grade of elementary school in Czechia and Slovakia, two middle European countries with common history and many similarities. Parents were recruited via cooperating schools that were instructed to ask all parents of children from the respected grades to participate in the study. Every participant created his own unique participant identification code. These codes were used to ensure that participants will remain anonymous, and the researchers can still pair both parents’ answers (if provided) to the same child [18].

Data collection took place during December 2020 and January 2021. It should be noted that in both Czechia and Slovakia, there were various restrictions related to the COVID-19 pandemic in place during this period. Parents were not remunerated for their participation. The participation consisted of completing the online survey, which took approximately 30–40 min.

### 2.2. Sample

In Czechia, 325 parents were recruited via six cooperating schools, which were selected from the Prague region based on convenience (past cooperation with the research team). In Slovakia, 84 parents were recruited via seven cooperating schools, which were selected based on (1) their location to represent various regions (west, middle, and east parts of the country) and (2) the willingness of school principals to distribute the questionnaire battery. Another 35 parents were recruited after direct approach by the researcher, thus bringing the total number of participants to 119 in this semi-convenient sample from Slovakia. In total, 103 participants were excluded: 100 based on missing values (more than 20% of missing values in the whole survey or any missing values in the part focused on media parenting) and 3 based on not having children in target grades. The final sample consisted of 341 Czech and Slovak parents. The detailed characteristics of the sample are presented in Table 1.

### 2.3. Measures

The survey consisted of socio-demographic questions, a media parenting scale (MEPA—see below), and children’s media/screen use. In relation to the COVID-19 pandemic situation, parents were asked whether their child is currently studying in school or distantly from their home.

#### 2.3.1. Media Parenting Scale for School-Aged Children (MEPA)

**Figure 1:**
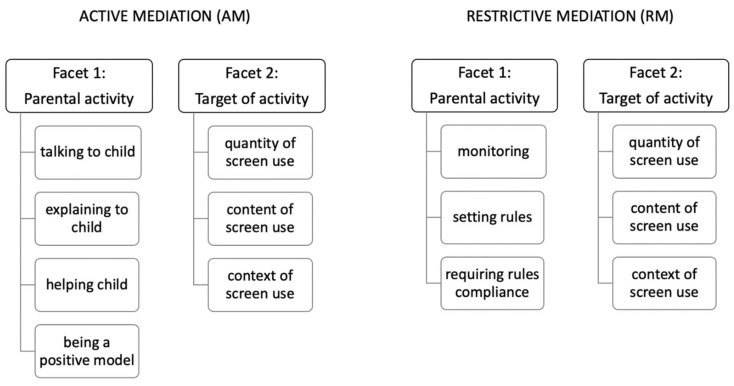
Facets of Active and Restrictive Mediation subscales.

As a first step of MEPA construction, we thoroughly analyzed the literature on media parenting (parental mediation) and also currently available measuring instruments. We identified over ten different instruments (scales) assessing media parenting, but only few of them were used more than once. The most frequently used instrument so far was the one developed by Livingstone and Helsper [7], and the second one developed within the EU Kids Online network [16]. We also analyzed other instruments [17,19,20,21,22,23,24]; based on this, we identified key facets (for the explanation of facet theory please see [25]) of active mediation (AM) and restrictive mediation (RM) and categories within each facet. Both AM and RM included two facets—“parental (regulative) activity” and “the target of this activity”. Categories for each facet are provided in Figure 1.

**Figure 2:**
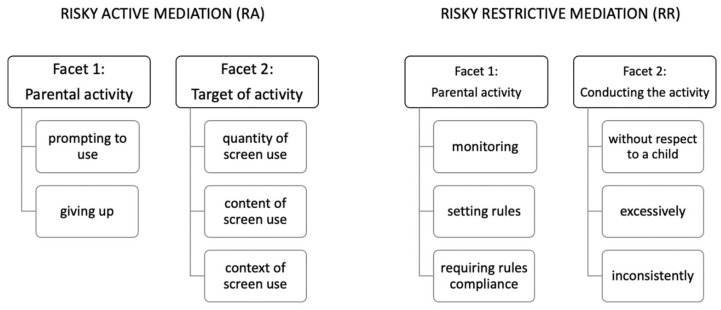
Facets of Risky Active and Risky Restrictive Mediation subscales.

Active and Restrictive Mediation are traditional concepts in media parenting literature. We added Risky Active and Risky Restrictive Mediation subscales in reflection of some previously constructed inventories, which mixed the “good” (presumably effective) and “bad” (presumably ineffective or even harmful) media parenting practices (facets are shown in Figure 2). Distinguishing between positive (effective) and negative (ineffective) practices is not usual within the area of media parenting, but it is well known in the area of general parenting (i.e., distinguishing between “positive” behavior and “negative” psychological control (e.g., love withdrawal, emotional manipulation etc.) [26]).

Items were systematically derived by combining categories across facets; for example, the AM item ‘I help my child to regulate the amount of time s/he spends using screens.’ is a combination of “helping” (facet 1—activity) and “quantity of screen use” (facet 2—target of the activity).

Using this procedure, we generated 36 items (12 items for AM, 9 items for RM, 6 items for RA, and 9 items for RR) constituting the MEPA-36 scale. The scale was constructed originally in Czech, but items were translated into Slovak and English (including back-translation procedure).

#### 2.3.2. Scoring of MEPA

Items are in the form of statements to which respondents (parents) express their level of agreement on a 5-point Likert scale (1 = totally untrue; 5 = totally true).

Four scores can be calculated as averages of relevant MEPA-36 items (a full list of items and their respective scales are in Table A1, Table A2 and Table A3):

- Active mediation (AM) reflects to what extent parents communicate with their child about screen use, how much they help him with different aspects of screen use (e.g., finding the right situations to use screens, finding the appropriate content etc.), and to what extent children can use a parent as a model for their own healthy screen use. Generally, the purpose of AM is to teach a child how to use technologies in a healthy and mindful way.
- Restrictive mediation (RM) reflects to what extent parents actively regulate their child’s use of screens by monitoring child’s screen activities, setting restrictive use, and to what extent parents insist that the child follows these rules. Generally, the purpose of RM is to externally regulate a child’s screen use to prevent her/him from overuse or inappropriate use of screens.
- Risky active mediation (RA) reflects to what extent parents voluntarily resign on active parenting of screen use and let the child handle screens by themselves and also to what extent they prompt their child to (over)use technologies. In general, RA practices may lead to excessive and uncontrolled use of screens.
- Risky restrictive mediation (RR) reflects to what extent parents overdo restrictive activities or conduct them in a harmful way; for example, by manipulating the child and by disregarding her/his privacy and self-regulation abilities. In general, parents using RR practices aim to have total control over a child’s use of screens and do not hesitate to use any means to reach it.

Similar scoring applies for the revised shortened scale MEPA-20, which consists of three subscales and provides three scores: active mediation, restrictive mediation and over-protective mediation (OP). OP consists of selected RR items (please see Table A4, Table A5 and Table A6).

#### 2.3.3. Child’s Screen Use

Two measures were developed to assess children’s screen use: screen time and risky screen use patterns.

Screen time was assessed via a parental report of *time spent in the average weekday* and *time spent in average weekend day* on four types of devices with screen: *portable screens* (smartphone or tablet), *gaming console*, *computer*, and *television*. Parents reported the time their child usually spends at each device using the scale: 0 (0 min per day), 1 (less than 30 min per day), 2 (30 min–1 h per day), 3 (1–2 h per day), 4 (2–3 h per day), 5 (3–4 h per day), and 6 (more than 4 h per day). To compute the estimated screen time for each child, we used the following procedure. First, we recalculated each response for each device to estimated time in minutes/hours: we used middle values for each interval (i.e., the response “2”, from 30 min to 1 h, we recalculated as 45 min (respectively 0.75 h); the response “3”, from 1 to 2 h, we recalculated as 90 min (respectively 1.5 h); the response “6”, more than 4 h, was calculated as “270 min” (respectively 4.5 h)). Second, we summed these recalculated estimates to provide the summative estimate of time spent on all devices. Third, we averaged the time spent on all devices during a typical working day and the time spent on all devices during a typical weekend day to estimate average daily screen time.

Risky screen use patterns were measured via seven items in which parents reported how often (never, about once a week, 2 to 3 times a week, every day, or almost every day) their child used a screen device in a way that is considered to be risky or harmful [27,28,29,30,31,32], namely.

- less than sixty minutes before bedtime
- while travelling
- while eating
- to calm down
- to fall asleep
- as a backdrop for other activities (e.g., playing, homework, etc.)

The inner consistency of scale was rather low (Cronbach α = 0.56; McDonald ω = 0.62), which is not unusual for scales with lower number of items and when analyzed using smaller samples.

### 2.4. Statistical Analyses

First, we performed psychometrical analyses in order to (1) assess the inner consistency of scales using Cronbach α and McDonald’s ω, and (2) examine the fit of MEPA using the confirmatory factor analysis. Two models were examined: the originally constructed 4-factor MEPA-36, and a revised and shortened 3-factor MEPA-20.

Second, we examined the relationships among MEPA subfactors and also between MEPA scores and other relevant variables, namely, child’s screen use. We presumed that high scores in positive media parenting scales (AM and RM) should—at least to some extent—prevent children from the development of an excessive/problematic use of screens. Contrary, we believed that high scores in risky media parenting scales (RA and RR), which reflect ineffective or even harmful parental behavior, may contribute to the development of the problematic use of screens either directly (in case of RA) or via damaging child–parent relationships, and via parental overprotection that does not allow the child to develop self-regulation of screen use (as in the case of RR). Moreover, we examined the relationship between MEPA and some sociodemographic characteristics of parents (age and gender) and children (age and gender); and also analyzed MEPA factors in relation to COVID-19 restrictions (as nearly half of the sample experienced distant, online schooling at the time of data collection).

### 2.5. Ethics

The study has been approved by an institutional ethical board of the Faculty of Education at Charles University. All participants provided the informed consent for participation.

## 3. Results

### 3.1. Socio-Demographic Characteristics of Final Sample

The age of participating parents ranged from 27 to 53 years (M = 40.3; SD = 4.47). Parents were mostly female (81.1%) with completed university education (56.9%). The age of children ranged from 72 months (i.e., 6 years) to 121 months (i.e., 10 years + 1 month) (M = 96.3 months, SD = 10.3 month). Children were 49% girls. Almost half of the children (46%) were distant (home) schooling at the time of data collection due to COVID-19 pandemic restrictions. More sample characteristics are provided in Table 1.

### 3.2. Psychometric Properties of MEPA

First, we assessed the inner consistency of the originally constructed scale (MEPA-36; full scale is in Appendix A). Then, we assessed the inner consistency of the revised (shortened) scale MEPA-20 (full scale is in Appendix B).

#### 3.2.1. MEPA-36

The inner consistency of Active Mediation (AM) subscale (12 items) was acceptable (Cronbach α = 0.82, McDonald ω = 0.83) and only one item (MP17) displayed an item-rest correlation lower than 0.30 (Table 2). The inner consistency of Restrictive Mediation (RM) subscale (9 items) was also acceptable (Cronbach α = 0.75, McDonald ω = 0.76) and no item displayed an item-rest correlation lower than 0.30 (Table 2). The inner consistency of Risky Active Mediation (RA) subscale (6 items) was low (Cronbach α = 0.58, McDonald ω = 0.59) and the majority of items did display weak-to-moderate item-rest correlations (Table 2). Moreover, we found that the means of RA items were very low, which meant that most parents did not report any RA practices. The inner consistency of Risky Restrictive Mediation (RR) subscale (9 items) was very low (Cronbach α = 0.42, McDonald ω = 0.48) with the majority of items displaying weak item-rest correlations; one item (MP34) displayed a negative correlation to the rest of the items (Table 2). As opposed to the RA subscale, in the case of RR, we identified a few items with a very high mean value (MP20 and MP30), meaning that parenting strategies expressed in these items were reported very frequently.

#### 3.2.2. MEPA-20

As the second step, we assessed the internal consistency of the revised scale MEPA-20. The revised version was developed to (a) shorten the scale to be better suited for the research use and more respondent-friendly, and (b) to address the suboptimal reliability measures of Risky Active and Risky Restrictive mediation subscales. MEPA-20 consists of 20 items divided into three subscales (active mediation, 8 items; restrictive mediation, 8 items; over-protective mediation (composed of selected Risky Restrictive Mediation items, 4 items). The Risky Active Mediation items were removed as it has been found that they were not relevant for media parenting of children in the target age.

The inner consistency of Active Mediation (AM) subscale (8 items) was acceptable (Cronbach α = 0.77, McDonald ω = 0.78) and no item displayed an item-rest correlation lower than 0.30 (Table 3). The inner consistency of Restrictive Mediation (RM) subscale (8 items) was acceptable (Cronbach α = 0.73, McDonald ω = 0.74) and no item displayed an item-rest correlation lower than 0.30 (Table 3). The inner consistency of Over-protective Mediation (OP) scale (4 items) was low (Cronbach α = 0.49, McDonald ω = 0.52), which is quite usual in short scales. Two items displayed an item-rest correlation lower than 0.30 (Table 3).

### 3.3. Factor Structure of MEPA

We assessed the model fit of two scales: (1) the original scale (MEPA-36) with four subscales and 36 items as described above, and (2) the revised scale (MEPA-20) with three subscales and 20 items.

Confirmatory factor analysis showed that the examined models differed in the model fit measures (Table 4). The better fit was found for MEPA-20. For MEPA-20, the ratio between χ**^2^** and degrees of freedom was 3.35, which was in the acceptable range (lower than 5.0) [33]. CFI and TLI values, which should be close to 1.0 or at least exceed 0.9, were found to be low for both examined models, but higher for MEPA-20. RMSEA values were found to be out of acceptable range—they should be lower than 0.07 [33]. On the other hand, in the case of MEPA-20, all items displayed significant loadings to their respective factors (*p* < 0.001). In the case of MEPA-36, there were three exceptions to this (items MP17, MP32, and MP36).

### 3.4. Score Distribution and Relationships between Variables

We found that mean values for Active and Restrictive Mediation (as measured by MEPA-20) were relatively high (almost 4 in a 1-to-5 response scale)—see Table 5. This suggested that parents reported the frequent use of media parenting practices (both active and restrictive ones). Contrary, over-protective practices were found less often. As for screen use measures, the estimated average screen time was relatively high for children of the target age (more than 3 h per day); however, this may be caused by restrictions related to the pandemic situation, which affected both the schooling (children were studying distantly from their homes, usually via online classrooms) and leisure activities.

As the next step, we examined the relationships between MEPA-20 scores and other relevant variables, namely, the child’s screen use measures: screen time and risky screen use patterns; results are presented in Table 5. We found significant positive and moderately strong associations among all MEPA scores (Active Mediation, Restrictive Mediation and Over-protective Mediation). The significant negative association was found between child’s screen use measures (screen time and risky screen use patterns) and Restrictive Mediation. Two screen use measures were positively and moderately associated with each other. Over-protective Mediation was weakly negatively associated with risky screen use patterns but not with screen time. We found close to zero associations between screen use and Active Mediation.

We also examined the relationships between MEPA and the sociodemographic characteristics of parents and children. We did not find any significant association between MEPA and the age of a parent or the age of a child, except for a small positive association between Active Mediation and child’s age (Pearson *r* = 0.13, *p* = 0.02). We found no differences in MEPA based on the child’s gender (*p*’s between 0.50 and 0.85; Cohen *d*’s < 0.1), but we found differences in MEPA based on the parent’s gender (i.e., between mothers (N = 275) and fathers (N = 64)). Mothers displayed significantly higher scores in Active Mediation (MD = 0.21, *p* = 0.02, and Cohen *d* = 0.33), Restrictive Mediation (MD = 0.18, *p* = 0.04, and Cohen *d* = 0.29), and Over-protective Mediation (MD = 0.22, *p* = 0.02, and Cohen *d* = 0.32).

Finally, we assessed the effect of distant schooling conditions on MEPA factors and other variables. We found no significant differences in MEPA between a distant (online) schooling group and a regular schooling group (*p*’s between 0.26 and 0.92; Cohen *d*’s between 0.01 and 0.13). However, we found differences between these two groups in screen use. The distant schooling group reported much higher daily screen time compared to the regular schooling group (MD = 1.53 h, *p* < 0.001, and Cohen *d* = 0.81) and also slightly higher risky screen use patterns (MD = 0.12, *p* = 0.013, and Cohen *d* = 0.27).

## 4. Discussion

In the field of media parenting, most measurement tools are self-generated for the purpose of one or a few studies. The construction and psychometrical quality of most such instruments is unknown [13]. Our aim was to develop a media parenting scale that would reflect current conceptualizations in the field, and, at the same time, would overcome most common problems of currently existing scales (e.g., mixing good and bad practices, focusing on only one device or one activity, and a lack of balance between active and restrictive approaches). Moreover, we aimed specifically for assessing practices of parents of primary school children (aged 6 to 10 years). Our scale (MEPA) has been constructed using the theory of facets [25]; the facets were derived from existing conceptualizations and measuring tools. The scale distinguished between active and restrictive approaches toward a child’s media/screen use and also between presumably positive (mediation) and potentially harmful practices (risky/over-protective mediation). The scale focuses on the use of screen-devices/media in general (rather than, for example, on gaming, the internet, or smartphone), which we believe reflects the way parents regulate screen-devices/media use in younger children—parents usually do not distinguish between various devices or activities but rather create regulative rules or guidelines for screen use generally [18].

Two scales were examined: the MEPA-36 with four subscales and 36 items and the MEPA-20 with three subscales and 20 items. The internal consistency was found to be acceptable in the case of the Active Mediation and Restrictive Mediation subscales both in MEPA-36 and MEPA-20. The inner consistency of subscales assessing the risky mediation practices (Risky Active Mediation and Risky Restrictive Mediation in case of MEPA-36 and Over-protective Mediation in case of MEPA-20) was found to be low, and these subscales need further testing. MEPA-20 displayed a better fit to the data compared to MEPA-36; however, most indices of model fit were out of recommended ranges for both MEPA-20 and MEPA-36. On the other hand, in the case of MEPA-20, all items displayed significant (*p* < 0.001) loadings to their respective subscale. Psychometrical properties of MEPA scale are comparable with those reported for currently existing media parenting scales for which psychometrical properties were published [19,23]. The lower model fit could be partially caused by the timing of data collection—almost half of participants provided data in the unprecedented situation of schools’ lockdown/family quarantine, related to the COVID-19 pandemic. We found no significant differences in reported media parenting practices (MEPA factors) between these participants and participants whose children attended school as usual. However, we found significant differences in the reported children’s screen use between these two groups. Further studies are needed to examine the model fit of MEPA.

We presumed that MEPA would be associated with a child’s screen use. We analyzed two screen use measures: screen time—a parental report of the summative time a child usually spends with all screen-based devices per day, and risky screen use patterns—a parental report of the frequency of screen use patterns that are considered to be harmful (e.g., using screens before sleeping or during meals). Restrictive Mediation was significantly and negatively associated with both screen use measures, but Active Mediation was not; this is in line with previous studies [11,13]. MEPA scores were not significantly associated with sociodemographic characteristics except for parental gender—mothers scored significantly higher than fathers in all three MEPA-20 subscales (Active Mediation, Restrictive Mediation, and Over-protective Mediation).

As the prevalence of screen-related problems in children is growing, the adequate response of the public health sector is necessary to empower their prevention and treatment. The family-based prevention of children’s problematic screen/media use is crucial because it is difficult to regulate daily screen use at the level of schools and other institutions [7]. While effective school-based prevention interventions addressing substance use are available [34,35,36], evidence-based interventions addressing screen/media use are lacking [37], and the ability of schools to address these risks is limited [38]. The first step to improve family-based prevention is to identify effective media parenting practices; this is only possible with appropriate instruments assessing these practices. Our study provided an easy-to-use and reliable scale for assessment of the media parenting practices of the parents of children aged 6 to 10 years. Most importantly, the scale enables assessment of parenting practices aimed at screen/media use in general (not only in the use of one individual screen device (e.g., smartphone) or one individual medium (e.g., games)). To the best of our knowledge, such a scale was missing for this age group. In addition, MEPA-20 assesses not only traditional concepts of active and restrictive mediation, but also over-protective mediation, which may be effective in the short term (when children are young), but problematic (counter-effective) in the long term, because it may prevent children from practicing self-regulation of their screen/media use.

The study has additional strengths, and also some limitations, worth noting. The main limitations are related to sampling. The sample size was rather small, and semi-convenient sampling was used. The study was designed as a feasibility study for a large project, in which the representative national samples will be used. Moreover, the survey was rather extensive, taking approximately 30–40 min to complete; this may have negatively affected the response rate. The above mentioned resulted in an unbalanced sample composition, consisting predominantly of well-educated female participants living in large cities. It is necessary to bear this limitation in mind when using study findings on the extent of media parenting practices and/or children’s screen use. Also, data was collected during COVID-19 pandemic, which may have influenced the screen use patterns in children. However, the focus of the study was not on the screen use but the media parenting practices, which did not seem to be affected by the pandemic situation. Finally, we did not use another previously published instrument for measuring media parenting alongside the MEPA, which meant we could not provide data on the congruent validity of MEPA. However, analysis of the existing instruments was the first step in developing the MEPA, and we believe that the MEPA reflected the key components of Active and Restrictive mediation well. As for risky mediation/over-protective mediation, we did not identify any previously published instruments that would explicitly measure these concepts.

## 5. Conclusions

The newly constructed scale MEPA is a reliable instrument, which can be used for assessing parenting practices related to the screen/media use (media parenting) of elementary school children (aged 6 to 10 years). The MEPA integrated previously constructed scales measuring media parenting and brought important distinctions between effective and potentially problematic (counter-effective) practices (i.e., over-protective mediation). This study may inform researchers designing studies on a broad range of screen/media activities in children (i.e., media/screen use in general, internet use, social networking, gaming, smartphone use etc.). Such studies are needed to identify effective media parenting strategies that may help parents and prevention professionals address children’s problematic screen use.

## Figures and Tables

**Table 1:** Characteristics of the sample.

Variable		Frequency
Age		M = 40.3 (SD = 4.47)
Nationality		
	Czech	65.4% (*n* = 223)
	Slovak	34.6% (*n* = 118)
Gender		
	Female	81.1% (*n* = 275)
	Male	18.9% (*n* = 64)
Education		
	Less than Highschool	2.9% (*n* = 10)
	Highschool graduate	26.4% (*n* = 90)
	University (BA degree)	8.8% (*n* = 30)
	University (MA degree or higher)	61.9% (*n* = 211)
Employment		
	Unemployed	5.3% (*n* = 18)
	Part-time job	18.2% (*n* = 62)
	Full-time job	65.1% (*n* = 222)
	Other	11.4% (*n* = 39)
Residence		
	Less than 999 inh.	6.2% (*n* = 21)
	1000–4999 inh.	12.6% (*n* = 43)
	5000–19,999 inh.	10.3% (*n* = 35)
	20,000–99,999 inh.	3.2% (*n* = 11)
	More than 100,000 inh.	67.7% (*n* = 231)
Family situation		
	Intact family	89.1% (*n* = 304)
	Single-parent family	3.8% (*n* = 13)
	Completed family	3.5% (*n* = 12)
	Alternating care	3.2% (*n* = 11)
	Other family	0.3% (*n* = 1)
Pandemic-related situation		
	At-home schooling	46.0% (*n* = 156)

**Table 2:** MEPA-36 Item Reliability Statistics.

Item	Mean	sd	Item-Rest Correlation	If Item Dropped	
				Cronbach’s α	McDonald’s ω
Active Mediation Items:					
MP1	3.86	1.155	0.5756	0.793	0.808
MP2	4.09	1.061	0.6027	0.791	0.807
MP4	4.19	1.057	0.45	0.805	0.82
MP5	4.12	1.044	0.396	0.809	0.824
MP7	4.15	0.979	0.5979	0.793	0.805
MP8	4.17	0.985	0.6427	0.789	0.801
MP10	3.71	1.106	0.3606	0.813	0.826
MP11	4.51	0.746	0.3685	0.811	0.826
MP13	3.74	1.192	0.6107	0.789	0.804
MP14	4.16	0.925	0.5712	0.796	0.809
MP16	3.33	1.228	0.4398	0.807	0.82
MP17	3.83	1.108	0.0783	0.837	0.843
Restrictive Mediation Items:					
MP19	4.58	0.675	0.448	0.734	0.739
MP21	3.96	1.108	0.492	0.72	0.732
MP23	3.42	1.118	0.34	0.746	0.757
MP25	4.3	0.808	0.395	0.737	0.748
MP27	3.83	1.11	0.49	0.721	0.732
MP29	3.55	1.272	0.441	0.731	0.744
MP31	3.96	0.957	0.414	0.733	0.744
MP33	4.06	0.989	0.503	0.72	0.731
MP35	3.55	1.169	0.424	0.733	0.746
Risky Active Mediation Items:					
MP3	1.5	0.941	0.274	0.558	0.565
MP6	1.41	0.914	0.375	0.514	0.525
MP9	1.36	0.866	0.382	0.513	0.523
MP12	1.45	0.737	0.284	0.554	0.568
MP15	2.02	1.042	0.299	0.55	0.556
MP18	2.12	1.032	0.321	0.539	0.545
Risky Restrictive Mediation Items:					
MP20	4.08	0.929	0.3089	0.345	0.408
MP22	2.55	1.353	0.218	0.369	0.448
MP24	2.92	1.126	0.2109	0.375	0.454
MP26	2.24	1.023	0.2148	0.375	0.446
MP28	2.95	1.139	0.3269	0.323	0.388
MP30	4.09	1.007	0.2099	0.377	0.442
MP32	2.01	0.914	0.1457	0.401	0.484
MP34	2.26	1.046	−0.1008	0.489	0.537
MP36	2.44	1.397	0.0746	0.443	0.499

**Table 3:** MEPA-20 Items Reliability Statistics.

Item	Mean	sd	Item-Rest Correlation	If Item Dropped	
				Cronbach’s α	McDonald’s ω
Active Mediation Items:					
MP1	3.86	1.155	0.485	0.743	0.756
MP2	4.12	1.044	0.32	0.77	0.781
MP3	4.17	0.985	0.608	0.723	0.735
MP4	3.71	1.106	0.347	0.767	0.777
MP6	4.51	0.746	0.365	0.762	0.776
MP7	3.74	1.192	0.637	0.713	0.727
MP8	4.16	0.925	0.585	0.729	0.737
MP9	3.33	1.228	0.46	0.749	0.762
Restrictive Mediation Items:					
MP11	3.96	1.108	0.47	0.698	0.705
MP12	3.42	1.118	0.327	0.727	0.734
MP13	4.3	0.808	0.373	0.718	0.724
MP14	3.83	1.11	0.488	0.694	0.698
MP16	3.55	1.272	0.446	0.704	0.713
MP17	3.96	0.957	0.41	0.71	0.715
MP18	4.06	0.989	0.494	0.695	0.7
MP19	3.55	1.169	0.427	0.707	0.716
Over-protective Mediation Items:					
MP5	2.55	1.35	0.221	0.495	0.532
MP10	2.92	1.13	0.235	0.464	0.584
MP15	2.24	1.02	0.306	0.406	0.539
MP20	2.95	1.14	0.406	0.305	0.391

**Table 4:** Model Fit Measures.

	χ^2^	df	CFI	TLI	RMSEA (90% CI)
MEPA-36	1730	588	0.644	0.619	0.076 (0.071–0.080)
MEPA-20	560	167	0.758	0.724	0.083 (0.076–0.091)

Note. χ ^2^ = χ^2^ after Satorra-Bentler correction; df, degrees of freedom; CFI, Comparative Fit Index; TLI, Tucker-Lewis Index; RMSEA, Root Mean Square Error of Approximation; CI, Confidence Interval.

**Table 5:** Means, Standard Deviations, and Pearson Correlation Coefficients for MEPA-20 Scores, Screen Time, and Risky Screen Use Patterns.

Variables		Mean	SD	Pearson Correlation Coefficient							
				1.		2.		3.		4.	
1.	Active Mediation	3.90	0.74								
2.	Restrictive Mediation	3.87	0.64	0.526	***						
3.	Over-protective Mediation	2.66	0.73	0.353	***	0.435	***				
4.	Screen time	3.37	2.03	0.023		−0.193	***	−0.044			
5.	Risky screen use patterns	0.76	0.44	0.016		−0.208	***	−0.129	*	0.456	***

Note. Screen time is in hours per average day. *** *p* < 0.001, * *p* < 0.05.

## Data Availability

Data for this study are available at https://osf.io/8nec4/ (accessed on 27 July 2021).

## Appendix A

**Table A1:** MEPA-36: English Version.

*Instructions*: Below you will find various statements that describe how parents can approach children’s use of screens. There are no right or wrong answers. Please rate how much each statement applies to you and your child.							
**Item Code**	**Subscale**	**Item**	**Response** ^1^				
MP1	AM	I chat with my child about time that s/he spends using screens.	1	2	3	4	5
MP2	AM	I explain to my child how much time s/he can spend using screens.	1	2	3	4	5
MP3	RA	I do not interfere with my child’s use of screens, because s/he knows best how to manage it.	1	2	3	4	5
MP4	AM	I help my child to regulate the amount of time s/he spends using screens.	1	2	3	4	5
MP5	AM	My child sees that I am able to regulate my screen time.	1	2	3	4	5
MP6	RA	I let my child use screens at will, because “what doesn’t kill you, makes you stronger”.	1	2	3	4	5
MP7	AM	I chat with my child about the content s/he consumes through screens (e.g., what videos s/he watches, games s/he plays, apps s/he uses, websites, texts, and pictures).	1	2	3	4	5
MP8	AM	I explain to my child which content is suitable for her/him (e.g., which videos, games, apps, websites, texts, and pictures)	1	2	3	4	5
MP9	RA	I convince my child that using screens is risk-free.	1	2	3	4	5
MP10	AM	I help my child to find suitable content (e.g., videos, games, apps, websites, texts, and pictures)	1	2	3	4	5
MP11	AM	In front of my child, I consume only such content (e.g., videos, games, apps, websites, texts, and pictures) that is also suitable for him/her.	1	2	3	4	5
MP12	RA	I prompt my child to use screens in excess (more than usual) if s/he needs to be entertained or calmed down.	1	2	3	4	5
MP13	AM	I chat with my child about how s/he uses screens (e.g., before going to bed, during meals, and during studying).	1	2	3	4	5
MP14	AM	I explain to my child in what situations the use of screens is in/appropriate.	1	2	3	4	5
MP15	RA	I prompt or let my child to use screens in excess (more than usual) if it is convenient for me (e.g., I need to concentrate on my work).	1	2	3	4	5
MP16	AM	I help my child to find suitable situations for watching/using screens.	1	2	3	4	5
MP17 *	AM	My child sees that I use screens even when inappropriate (e.g., during meals).	1	2	3	4	5
MP18	RA	I persuade my child to use screens (technologies) to keep up with times.	1	2	3	4	5
MP19	RM	I notice how much time my child spends using screens.	1	2	3	4	5
MP20	RR	I am the one who sets rules, my child is only supposed to obey them.	1	2	3	4	5
MP21	RM	We have agreed rules about screen time.	1	2	3	4	5
MP22	RR	I secretly check my child’s screen activities (when and what my child was watching or what apps s/he was using).	1	2	3	4	5
MP23	RM	I do not let my child use screens longer than agreed.	1	2	3	4	5
MP24	RR	I let my child know that watching/using screens is a waste of time.	1	2	3	4	5
MP25	RM	I notice what content my child consumes through screens (e.g., what videos s/he watches, what games s/he plays, what apps s/he uses, and what text and pictures).	1	2	3	4	5
MP26	RR	In our family, we set rules, which almost never allow children to use screens for entertainment.	1	2	3	4	5
MP27	RM	We have agreed rules about what my child may and may not watch/do on screens (e.g., which videos, games, apps, texts, and pictures).	1	2	3	4	5
MP28	RR	I constantly check my child’s screen activities.	1	2	3	4	5
MP29	RM	I do not let my child consume other content than agreed.	1	2	3	4	5
MP30	RR	When my child uses screens differently from what is allowed, I will immediately go for force quit.	1	2	3	4	5
MP31	RM	I notice in which situations my child watches and uses screens.	1	2	3	4	5
MP32	RR	I change rules about the use of screens at will according to what is currently most convenient for me.	1	2	3	4	5
MP33	RM	We have rules specifying situations in which my child is or is not allowed to watch/use screens.	1	2	3	4	5
MP34	RR	I monitor my child’s screen activities only when it crosses my mind to do so.	1	2	3	4	5
MP35	RM	I do not let my child use screens in other than agreed situations.	1	2	3	4	5
MP36	RR	When my child uses a screen inappropriately, I sometimes do intervene and sometimes don’t.	1	2	3	4	5

^1^ 1 (totally untrue), 2 (rather untrue), 3 (neither true nor untrue), 4 (rather true), and 5 (totally true). * reverse coded item. Scoring instructions: The score of Active Mediation (AM) compute as an average of responses on items MP1, MP2, MP4, MP5, MP7, MP8, MP10, MP11, MP13, MP14, MP16, and MP17. Note that MP17 is reverse coded (i.e., you need to recompute the response as follows: 1 = 5, 2 = 4, 3 = 3, 4 = 2, and 5 = 1). The score of Restrictive Mediation (RM) compute as an average of responses on items MP19, MP21, MP23, MP25, MP27, MP29, MP31, MP33, and MP35. The score of Risky Active Mediation (RA) compute as an average of responses on items MP3, MP6, MP9, MP12, MP15, and MP18. The score of Risky Restrictive Mediation (RR) compute as an average of responses on items MP20, MP22, MP24, MP26, MP28, MP30, MP32, MP34, and MP36.

**Table A2:** MEPA-36: Czech Version.

*Instrukce*: Níže najdete různé výroky, které popisují, jak mohou rodiče přistupovat k dětskému užívání obrazovek. Neexistují správné a špatné odpovědi. Prosíme, ohodnoťte, nakolik každý z výroků platí pro vás a vaše dítě.							
**Item Code**	**Subscale**	**Item**	**Response** ^1^				
MP1	AM	S dítětem si povídám o tom, kolik času tráví s obrazovkami.	1	2	3	4	5
MP2	AM	Dítěti vysvětluji, kolik času může strávit s obrazovkami.	1	2	3	4	5
MP3	RA	Nezasahuji dítěti do používání obrazovek, protože ono samo ví nejlíp, jak si s nimi poradit.	1	2	3	4	5
MP4	AM	Pomáhám dítěti hlídat čas strávený s obrazovkami.	1	2	3	4	5
MP5	AM	Dítě vidí, že dokážu kontrolovat dobu, kterou strávím s obrazovkami.	1	2	3	4	5
MP6	RA	Dítě nechávám používat obrazovky podle jeho uvážení ve smyslu přísloví “co tě nezabije, to tě posílí”.	1	2	3	4	5
MP7	AM	S dítětem si povídám o tom, co prostřednictvím obrazovky konzumuje (např. na jaká videa se dívá, jaké hry hraje, jaké aplikace používá, jaké webové stránky, jaké články a obrázky).	1	2	3	4	5
MP8	AM	Dítěti vysvětluji, jaký obsah je pro něj vhodný (např. jaká videa, jaké hry, jaké aplikace, jaké webové stránky, jaké články a obrázky).	1	2	3	4	5
MP9	RA	Přesvědčuji dítě, že užívání obrazovek je bez rizika.	1	2	3	4	5
MP10	AM	Dítěti pomáhám vyhledávat vhodný obsah (např. videa, hry, aplikace, webové stránky, články a obrázky).	1	2	3	4	5
MP11	AM	Před dítětem konzumuji pouze takový obsah (např. videa, hry, aplikace, webové stránky, články a obrázky), který je pro něj/ni vhodný.	1	2	3	4	5
MP12	RA	Dítě povzbuzuji k používání obrazovek (i nad obvyklou míru), když potřebuje zabavit nebo uklidnit.	1	2	3	4	5
MP13	AM	S dítětem si povídám o tom, jakým způsobem používá obrazovky (např. před spaním, při učení, při jídle...).	1	2	3	4	5
MP14	AM	Dítěti vysvětluji, ve kterých situacích používání obrazovek je a není vhodné.	1	2	3	4	5
MP15	RA	Dítě pobízím k používání obrazovek (i nad obvyklou míru), pokud je to pro mě výhodné (např. potřebuji klid na práci).	1	2	3	4	5
MP16	AM	Dítěti pomáhám najít vhodné situace, ve kterých může používat obrazovky.	1	2	3	4	5
MP17 *	AM	Dítě je svědkem toho, že obrazovky používám i v nevhodných situacích (např. během jídla).	1	2	3	4	5
MP18	RA	Dítě povzbuzuji k používání obrazovek (technologií), aby drželo krok s dobou.	1	2	3	4	5
MP19	RM	Všímám si, kolik času dítě tráví s obrazovkami.	1	2	3	4	5
MP20	RR	Pravidla používání obrazovek určuji já, dítě je má pouze dodržovat.	1	2	3	4	5
MP21	RM	Máme dohodnutá pravidla, kolik času dítě stráví s obrazovkami.	1	2	3	4	5
MP22	RR	Tajně kontroluji, co a kdy dítě na obrazovce sledovalo nebo jaké aplikace používalo.	1	2	3	4	5
MP23	RM	Nenechám dítě s obrazovkou déle, než máme dohodnuto.	1	2	3	4	5
MP24	RR	Dítěti dávám najevo, že používat obrazovky je podle mě ztráta času.	1	2	3	4	5
MP25	RM	Všímám si toho, co dítě prostřednictvím obrazovky konzumuje (např. na jaká videa se dívá, jaké hry hraje, jaké aplikace používá, jaké články a obrázky).	1	2	3	4	5
MP26	RR	Pravidla nastavujeme tak, aby dítě nepoužívalo obrazovky pro zábavu téměř vůbec.	1	2	3	4	5
MP27	RM	Máme dohodnutá pravidla, čemu se dítě na obrazovce smí a nesmí věnovat (kterým hrám, videím, aplikacím, textům, obrázkům...).	1	2	3	4	5
MP28	RR	Neustále kontroluji, čemu se dítě na obrazovce věnuje.	1	2	3	4	5
MP29	RM	Nenechám dítě konzumovat jiný obsah než ten, na kterém jsme se domluvili.	1	2	3	4	5
MP30	RR	Kdykoliv mé dítě používá obrazovku jinak, než má dovoleno, okamžitě ho donutím, aby přestalo.	1	2	3	4	5
MP31	RM	Všímám si, v jakých situacích dítě sleduje a používá obrazovky.	1	2	3	4	5
MP32	RR	Pravidla měním podle toho, jak se mi to v aktuální situaci nejvíc hodí.	1	2	3	4	5
MP33	RM	Máme pravidla, v jakých situacích dítě smí a nesmí sledovat a používat obrazovky.	1	2	3	4	5
MP34	RR	Sleduji, co dítě na obrazovce dělá, jen když si na to zrovna vzpomenu.	1	2	3	4	5
MP35	RM	Nenechám dítě sledovat nebo používat obrazovky mimo dohodnuté situace.	1	2	3	4	5
MP36	RR	Pokud dítě používá obrazovky nevhodně, někdy zasáhnu, jindy ne.	1	2	3	4	5

^1^ 1 (vůbec neplatí), 2 (spíše neplatí), 3 (tak napůl), 4 (spíše platí), 5 (rozhodně platí). * reverzně kódovaná položka. Instrukce pro skórování: Skór Aktivní Mediace (AM) vypočtěte jako průměr odpovědí na položky MP1, MP2, MP4, MP5, MP7, MP8, MP10, MP11, MP13, MP14, MP16, a MP17. Mějte na paměti, že položka MP17 je opačně kódovaná (tj. je třeba jí přepočítat následujícím způsobem: 1 = 5, 2 = 4, 3 = 3, 4 = 2, and 5 = 1). Skór Restriktivní Mediace (RM) vypočtěte jako průměr odpovědí na položky MP19, MP21, MP23, MP25, MP27, MP29, MP31, MP33, a MP35. Skór Rizikové Aktivní Mediace (RA) vypočtěte jako průměr odpovědí na položky MP3, MP6, MP9, MP12, MP15, a MP18. Skór Rizikové Restriktivní Mediace (RR) vypočtěte jako průměr odpovědí na položky MP20, MP22, MP24, MP26, MP28, MP30, MP32, MP34, a MP36.

**Table A3:** MEPA-36: Slovak version.

*Inštrukcia:* V tejto časti sú rôzne výroky, ktoré hovoria, ako môžu rodičia pristupovať k detskému užívaniu obrazoviek. Neexistujú správne a zlé odpovede. Prosíme, ohodnoťte, nakoľko každý z výrokov platí pre vás a vaše dieťa.							
**Item Code**	**Subscale**	**Item**	**Response** ^1^				
MP1	AM	S dieťaťom sa rozprávam o tom, koľko času tráví s obrazovkami.	1	2	3	4	5
MP2	AM	Dieťaťu vysvetľujem, koľko času môže stráviť s obrazovkami.	1	2	3	4	5
MP3	RA	Nezasahujem dieťaťu do používania obrazoviek, pretože ono samo vie najlepšie, ako si s nimi poradit.	1	2	3	4	5
MP4	AM	Pomáhám dieťaťu strážiť si čas, strávený s obrazovkami.	1	2	3	4	5
MP5	AM	Dieťa vidí, že dokážem kontrolovať dobu, ktorú strávim s obrazovkami.	1	2	3	4	5
MP6	RA	Dieťa nechávam používať obrazovky podľa jeho uváženia v zmysle príslovia „čo ťa nezabije, to ťa posilní“.	1	2	3	4	5
MP7	AM	S dieťaťom sa rozprávam o tom, čo prostredníctvom obrazovky konzumuje (napr. na aké videa sa pozerá, aké hry hraje, aké aplikácie používá, aké webové stránky, aké články a obrázky).	1	2	3	4	5
MP8	AM	Dieťaťu vysvetľujem, aký obsah je pre neho vhodný (napr. na aké videá sa pozerá, aké hry hraje, aké aplikácie používá, aké webové stránky, aké články a obrázky).	1	2	3	4	5
MP9	RA	Presviedčam dieťa, že užívanie obrazoviek je bez rizika. (Presvedčujem dieťa, že pri používaní obrazoviek nehrozí žiadne nebezpečenstvo)	1	2	3	4	5
MP10	AM	Dieťaťu pomáhám vyhľadávať vhodný obsah (napr. videá, hry, aplikácie, webové stránky, články a obrázky).	1	2	3	4	5
MP11	AM	Pred dieťaťom konzumujem iba taký obsah (napr. videá, hry, aplikácie, webové stránky, články a obrázky), ktorý je pre neho vhodný.	1	2	3	4	5
MP12	RA	Dieťa povzbudzujem k používaniu obrazoviek (i nad obvyklú mieru), keď ho potrebujem zabaviť alebo utíšiť.	1	2	3	4	5
MP13	AM	S dieťaťom sa rozprávame o tom, akým spôsobom používa obrazovky (napr. pred spaním, pri učení, pri jedle...).	1	2	3	4	5
MP14	AM	Dieťaťu vysvetľujem, v ktorých situáciách je používanie obrazoviek vhodné a v ktorých nie.	1	2	3	4	5
MP15	RA	Dieťa povzbudzujem k používaniu obrazoviek (i nad obvyklú mieru), ak je to pre mňa výhodné (napr. potrebujem kľud na prácu).	1	2	3	4	5
MP16	AM	Dieťaťu pomáham nájsť vhodné situácie, v ktorých môže používať obrazovky.	1	2	3	4	5
MP17 *	AM	Dieťa je svedkom toho, že obrazovky používam aj v nevhodných situáciách (napr. počas jedla).	1	2	3	4	5
MP18	RA	Dieťa povzbudzujem k používaniu obrazoviek (technológií), aby držalo krok s dobou.	1	2	3	4	5
MP19	RM	Všímám si, koľko času dieťa trávi s obrazovkami.	1	2	3	4	5
MP20	RR	Pravidlá používania obrazoviek určujem ja, dieťa ich má iba dodržiavať.	1	2	3	4	5
MP21	RM	Máme dohodnuté pravidlá, koľko času má dieťa strávíť s obrazovkami.	1	2	3	4	5
MP22	RR	Tajne kontrolujem, čo a kedy dieťa na obrazovke sledovalo alebo aké aplikácie používalo.	1	2	3	4	5
MP23	RM	Nenechám dieťa s obrazovkou dlhšie, než máme dohodnuté.	1	2	3	4	5
MP24	RR	Dieťaťu dávam najavo, že používať obrazovky je podľa mňa strata času.	1	2	3	4	5
MP25	RM	Všímám si to, čo dieťa prostredníctvom obrazovky konzumuje (napr. na aké videá sa pozerá, aké hry hrá, aké aplikácie používa, aké články a obrázky).	1	2	3	4	5
MP26	RR	Pravidlá nastavujeme tak, aby dieťa nepoužívalo obrazovky pre zábavu takmer vôbec.	1	2	3	4	5
MP27	RM	Máme dohodnuté pravidlá, čomu sa dieťa na obrazovke smie a nesmie venovať (ktorým hrám, videám, aplikáciám, textom, obrázkom...).	1	2	3	4	5
MP28	RR	Neustále kontrolujem, čomu sa dieťa na obrazovke venuje.	1	2	3	4	5
MP29	RM	Nenechám dieťa konzumovať iný obsah než ten, na ktorom sme sa dohodli.	1	2	3	4	5
MP30	RR	Ak dieťa poruší pravidlá, ako má používať obrazovku, trvám na tom, aby ju používať okamžite prestalo (ihneď zariadenie vypnem alebo mu ho odeberiem).	1	2	3	4	5
MP31	RM	Všímám si, v ktorých situáciách dieťa sleduje a používa obrazovky.	1	2	3	4	5
MP32	RR	Pravidlá mením podľa toho, ako sa mi to v aktuálnej situácii najviac hodí.	1	2	3	4	5
MP33	RM	Máme pravidlá, v akých situáciách dieťa smie a nesmie sledovať a používať obrazovky.	1	2	3	4	5
MP34	RR	Sledujem, čo dieťa na obrazovke robí, iba vtedy, keď si na to zrovna spomeniem.	1	2	3	4	5
MP35	RM	Nenechám dieťa sledovať alebo používať obrazovky mimo dohodnuté situácie.	1	2	3	4	5
MP36	RR	Pokiaľ dieťa používá obrazovky nevhodne, niekedy zasiahnem, inokedy nie.	1	2	3	4	5

^1^ 1 (vôbec neplatí), 2 (skôr neplatí) 3 (tak z polovice), 4 (skôr platí), 5 (rozhodne platí). * reverzne kódovaná položka. Inštrukcie pre skórovanie: Skóre Aktívna Mediácia (AM) vypočítame ako priemer odpovedí na položky MP1, MP2, MP4, MP5, MP7, MP8, MP10, MP11, MP13, MP14, MP16, a MP17. Majte na pamäti, že položka MP17 je opačne kódovaná (tj. je treba ju prepočítať následujícím spôsobom: 1 = 5, 2 = 4, 3 = 3, 4 = 2, and 5 = 1). Skóre Reštriktívna Mediácia (RM) vypočítáme ako priemer odpovedí na položky MP19, MP21, MP23, MP25, MP27, MP29, MP31, MP33, a MP35. Skóre Riziková Aktívna Mediácia (RA) vypočítame ako priemer odpovedí na položky MP3, MP6, MP9, MP12, MP15, a MP18. Skóre Riziková Reštriktívna Mediácia (RR) vypočítame ako priemer odpovedí na položky MP20, MP22, MP24, MP26, MP28, MP30, MP32, MP34, a MP36.

## Appendix B

**Table A4:** MEPA-20: English Version.

*Instructions*: Below you will find various statements that describe how parents can approach children’s use of screens. There are no right or wrong answers. Please rate how much each statement applies to you and your child.							
**Item Code**	**Subscale**	**Item**	**Response** ^1^				
MP1	AM	I chat with my child about time that s/he spends using screens.	1	2	3	4	5
MP2	AM	My child sees that I am able to regulate my screen time.	1	2	3	4	5
MP3	AM	I explain to my child which content is suitable for her/him (e.g., which videos, games, apps, websites, texts, and pictures)	1	2	3	4	5
MP4	AM	I help my child to find suitable content (e.g., videos, games, apps, websites, texts and pictures)	1	2	3	4	5
MP5	OP	I secretly check my child’s screen activities (when and what my child was watching or what apps s/he was using).	1	2	3	4	5
MP6	AM	In front of my child, I consume only such content (e.g., videos, games, apps, websites, texts, and pictures) that is also suitable for him/her.	1	2	3	4	5
MP7	AM	I chat with my child about how s/he uses screens (e.g., before going to bed, during meals, and during studying).	1	2	3	4	5
MP8	AM	I explain to my child in what situations the use of screens is in/appropriate.	1	2	3	4	5
MP9	AM	I help my child to find suitable situations for watching/using screens.	1	2	3	4	5
MP10	OP	I let my child know that watching/using screens is a waste of time.	1	2	3	4	5
MP11	RM	We have agreed rules about screen time.	1	2	3	4	5
MP12	RM	I do not let my child use screens longer than agreed.	1	2	3	4	5
MP13	RM	I notice what content my child consumes through screens (e.g., what videos s/he watches, games s/he plays, apps s/he uses, text, and pictures).	1	2	3	4	5
MP14	RM	We have agreed rules about what my child may and may not watch on/do on screens (e.g., which videos, games, apps, texts, and pictures).	1	2	3	4	5
MP15	OP	I constantly check my child’s screen activities.	1	2	3	4	5
MP16	RM	I do not let my child consume other content than agreed.	1	2	3	4	5
MP17	RM	I notice in which situations my child watches and uses screens.	1	2	3	4	5
MP18	RM	We have rules specifying situations in which my child is or is not allowed to watch/use screens.	1	2	3	4	5
MP19	RM	I do not let my child use screens in other than agreed situations.	1	2	3	4	5
MP20	OP	In our family, we set rules which almost never allow children to use screens for entertainment.	1	2	3	4	5

^1^ 1 (totally untrue), 2 (rather untrue), 3 (neither true nor untrue), 4 (rather true), and 5 (totally true). Scoring instructions: The score of Active Mediation (AM) compute as an average of responses on items MP1, MP2, MP3, MP4, MP6, MP7, MP8, MP9. The score of Restrictive Mediation (RM) compute as an average of responses on items MP11, MP12, MP13, MP14, MP16, MP17, MP18, and MP19. The score of Over-protective Mediation (OP) compute as an average of responses on items MP5, MP10, MP15, and MP20.

**Table A5:** MEPA-20: Czech version.

*Instrukce*: Níže najdete různé výroky, které popisují, jak mohou rodiče přistupovat k dětskému užívání obrazovek. Neexistují správné a špatné odpovědi. Prosíme, ohodnoťte, nakolik každý z výroků platí pro vás a vaše dítě.							
**Item Code**	**Subscale**	**Item**	**Response** ^1^				
MP1	AM	S dítětem si povídám o tom, kolik času tráví s obrazovkami.	1	2	3	4	5
MP2	AM	Dítě vidí, že dokážu kontrolovat dobu, kterou strávím s obrazovkami.	1	2	3	4	5
MP3	AM	Dítěti vysvětluji, jaký obsah je pro něj vhodný (např. jaká videa, jaké hry, jaké aplikace, jaké webové stránky, jaké články a obrázky).	1	2	3	4	5
MP4	AM	Dítěti pomáhám vyhledávat vhodný obsah (např. videa, hry, aplikace, webové stránky, články a obrázky).	1	2	3	4	5
MP5	OP	Tajně kontroluji, co a kdy dítě na obrazovce sledovalo nebo jaké aplikace používalo.	1	2	3	4	5
MP6	AM	Před dítětem konzumuji pouze takový obsah (např. videa, hry, aplikace, webové stránky, články a obrázky), který je pro něj/ni vhodný.	1	2	3	4	5
MP7	AM	S dítětem si povídám o tom, jakým způsobem používá obrazovky (např. před spaním, při učení, při jídle...).	1	2	3	4	5
MP8	AM	Dítěti vysvětluji, ve kterých situacích používání obrazovek je a není vhodné.	1	2	3	4	5
MP9	AM	Dítěti pomáhám najít vhodné situace, ve kterých může používat obrazovky.	1	2	3	4	5
MP10	OP	Dítěti dávám najevo, že používat obrazovky je podle mě ztráta času.	1	2	3	4	5
MP11	RM	Máme dohodnutá pravidla, kolik času dítě stráví s obrazovkami.	1	2	3	4	5
MP12	RM	Nenechám dítě s obrazovkou déle, než máme dohodnuto.	1	2	3	4	5
MP13	RM	Všímám si toho, co dítě prostřednictvím obrazovky konzumuje (např. na jaká videa se dívá, jaké hry hraje, jaké aplikace používá, jaké články a obrázky).	1	2	3	4	5
MP14	RM	Máme dohodnutá pravidla, čemu se dítě na obrazovce smí a nesmí věnovat (kterým hrám, videím, aplikacím, textům, obrázkům...).	1	2	3	4	5
MP15	OP	Neustále kontroluji, čemu se dítě na obrazovce věnuje.	1	2	3	4	5
MP16	RM	Nenechám dítě konzumovat jiný obsah než ten, na kterém jsme se domluvili.	1	2	3	4	5
MP17	RM	Všímám si, v jakých situacích dítě sleduje a používá obrazovky.	1	2	3	4	5
MP18	RM	Máme pravidla, v jakých situacích dítě smí a nesmí sledovat a používat obrazovky.	1	2	3	4	5
MP19	RM	Nenechám dítě sledovat nebo používat obrazovky mimo dohodnuté situace.	1	2	3	4	5
MP20	OP	Pravidla nastavujeme tak, aby dítě nepoužívalo obrazovky pro zábavu téměř vůbec.	1	2	3	4	5

^1^ 1 (vůbec neplatí), 2 (spíše neplatí), 3 (tak napůl), 4 (spíše platí), 5 (rozhodně platí). Instrukce pro skórování: Skór Aktivní Mediace (AM) vypočtěte jako průměr odpovědí na položky MP1, MP2, MP3, MP4, MP6, MP7, MP8, a MP9. Skór Restriktivní Mediace (RM) vypočtěte jako průměr odpovědí na položky MP11, MP12, MP13, MP14, MP16, MP17, MP18, a MP19. Skór Hyperprotektivní Mediace (OP) vypočtěte jako průměr odpovědí na položky MP5, MP10, MP15, a MP20.

**Table A6:** MEPA-20: Slovak version.

*Instrukce*: Níže najdete různé výroky, které popisují, jak mohou rodiče přistupovat k dětskému užívání obrazovek. Neexistují správné a špatné odpovědi. Prosíme, ohodnoťte, nakolik každý z výroků platí pro vás a vaše dítě.							
**Item Code**	**Subscale**	**Item**	**Response** ^1^				
MP1	AM	S dieťaťom sa rozprávam o tom, koľko času tráví s obrazovkami.	1	2	3	4	5
MP2	AM	Dieťa vidí, že dokážem kontrolovať dobu, ktorú strávim s obrazovkami.	1	2	3	4	5
MP3	AM	Dieťaťu vysvetľujem, aký obsah je pre neho vhodný (napr. na aké videá sa pozerá, aké hry hraje, aké aplikácie používá, aké webové stránky, aké články a obrázky).	1	2	3	4	5
MP4	AM	Dieťaťu pomáham vyhľadávať vhodný obsah (napr. videá, hry, aplikácie, webové stránky, články a obrázky).	1	2	3	4	5
MP5	OP	Tajne kontrolujem, čo a kedy dieťa na obrazovke sledovalo alebo aké aplikácie používalo.	1	2	3	4	5
MP6	AM	Pred dieťaťom konzumujem iba taký obsah (napr. videá, hry, aplikácie, webové stránky, články a obrázky), ktorý je pre neho vhodný.	1	2	3	4	5
MP7	AM	S dieťaťom sa rozprávame o tom, akým spôsobom používa obrazovky (napr. pred spaním, pri učení, pri jedle...).	1	2	3	4	5
MP8	AM	Dieťaťu vysvetľujem, v ktorých situáciách je používanie obrazoviek vhodné a v ktorých nie.	1	2	3	4	5
MP9	AM	Dieťaťu pomáham nájsť vhodné situácie, v ktorých môže používať obrazovky.	1	2	3	4	5
MP10	OP	Dieťaťu dávam najavo, že používať obrazovky je podľa mňa strata času.	1	2	3	4	5
MP11	RM	Máme dohodnuté pravidlá, koľko času má dieťa strávíť s obrazovkami.	1	2	3	4	5
MP12	RM	Nenechám dieťa s obrazovkou dlhšie, než máme dohodnuté.	1	2	3	4	5
MP13	RM	Všímám si to, čo dieťa prostredníctvom obrazovky konzumuje (napr. na aké videá sa pozerá, aké hry hrá, aké aplikácie používa, aké články a obrázky).	1	2	3	4	5
MP14	RM	Máme dohodnuté pravidlá, čomu sa dieťa na obrazovke smie a nesmie venovať (ktorým hrám, videám, aplikáciám, textom, obrázkom...).	1	2	3	4	5
MP15	OP	Neustále kontrolujem, čomu sa dieťa na obrazovke venuje.	1	2	3	4	5
MP16	RM	Nenechám dieťa konzumovať iný obsah než ten, na ktorom sme sa dohodli.	1	2	3	4	5
MP17	RM	Všímám si, v ktorých situáciách dieťa sleduje a používa obrazovky.	1	2	3	4	5
MP18	RM	Máme pravidlá, v akých situáciách dieťa smie a nesmie sledovať a používať obrazovky.	1	2	3	4	5
MP19	RM	Nenechám dieťa sledovať alebo používať obrazovky mimo dohodnuté situácie.	1	2	3	4	5
MP20	OP	Pravidlá nastavujeme tak, aby dieťa nepoužívalo obrazovky pre zábavu takmer vôbec.	1	2	3	4	5

^1^ 1 (vôbec neplatí), 2 (skôr neplatí) 3 (tak z polovice), 4 (skôr platí), 5 (rozhodne platí). Inštrukcie pre skórovanie: Skóre Aktívna Mediácia (AM) vypočítame ako priemer odpovedí na položky MP1, MP2, MP3, MP4, MP6, MP7, MP8, a MP9. Skóre Reštriktívna Mediácia (RM) vypočítame ako priemer odpovedí na položky MP11, MP12, MP13, MP14, MP16, MP17, MP18, a MP19. Skóre Hyperprotektívna Mediácia (OP) vypočítame ako priemer odpovedí na položky MP5, MP10, MP15, a MP20.
